# Video-endoscopic inguinal lymphadenectomy (VEIL) oncological and surgical benefits compared to open inguinal lymph node dissection (ILND)

**DOI:** 10.22514/jomh.2024.106

**Published:** 2024-07-30

**Authors:** Fayssal Alqudrah, Rachel Passarelli, Jennifer Sykes, Raeesa Islam, Kevin Chua, Saum Ghodoussipour

**Affiliations:** 1 Division of Urology, Robert Wood Johnson Medical School, New Brunswick, NJ 08901, USA; 2 Section of Urologic Oncology, Rutgers Cancer Institute of New Jersey, New Brunswick, NJ 08901, USA

**Keywords:** Penile cancer, Minimally invasive, Video-endoscopic inguinal lymphadenectomy, Robotic, Inguinal lymphadenectomy

## Abstract

Penile cancer accounts for about 1% of all male cancer diagnoses in the United States. Regional lymph node involvement is strongly correlated with overall outcomes, and as such the procedure of inguinal lymph node dissection (ILND) is imperative to the diagnostic and oncologic outcomes for these patients. The current gold standard of open ILND presents challenges in mitigating detrimental postoperative sequalae such as wound complications and lymphedema, without compromising oncologic outcomes. There has been a growing interest and shift in minimally invasive (MIS) approaches to tackle the challenges seen in the open approach to ILND. Several different minimally invasive techniques such as laparoscopic video-endoscopic inguinal lymphadenectomy (VEIL) and robotic-assisted VEIL approaches have been explored and described in the literature. A systematic literature search of PubMed and Medline (OVID) literature review was performed to assess outcomes in MIS approaches to ILND in comparison to traditional open approach. Key words included penile cancer/penile neoplasms, minimally-invasive procedures, robotics, video-endoscopic, robotic-assisted, inguinal lymphadenectomy, and inguinal lymph node dissections. Studies show that MIS approaches to ILND have potential to reduce high-grade postoperative complications, operative time, and hospital stay while ensuring oncologic outcomes. Despite the learning curve associated with MIS ILND, preliminary data does suggest favorable outcomes. Prospective, randomized trials are needed to reveal the full benefit of MIS ILND compared to open ILND.

## 1. Introduction

In North America and Europe, penile cancer is rare with fewer than 1 in 100,000 males diagnosed each year accounting for less than 1% of cancers in men in the United States. However, the 5-year relative survival rate for penile cancer that has spread to regional lymph nodes is 51% and can drop to 9% if the cancer spreads to distant body parts. Regional lymph node involvement is strongly associated with the prognosis and survival of patients with penile cancer. Safe and complete inguinal lymphadenectomy is vital for the removal of lymph-node metastases and improving outcomes for patients [1–4]. Despite the benefits and necessity of inguinal lymph node dissection (ILND), the procedure is associated with certain risks. Open ILND has been, and still remains the gold standard for the prophylactic management of patients with high risk of nodal spread and for the treatment of palpable inguinal lymphadenopathy [5, 6]. However, open ILND is associated with a high incidence of wound complications and overall morbidity with prevalence of complications reaching up to 90% [7]. Other sequalae include lymphocele, femoral vessel and nerve injury, lymphedema, skin flap necrosis and recurrence [8, 9]. To overcome these challenges, minimally invasive surgical (MIS) approaches have emerged including laparoscopic video-endoscopic inguinal lymphadenectomy (VEIL) and robotic-assisted VEIL (R-VEIL) as alternatives to traditional open ILND. Although VEIL has limitations that have prevented universal adoption, recent studies suggest promising outcomes related to wound complications and overall morbidity compared to open ILND. Here we provide a comprehensive review of current literature and discuss the surgical technique of minimally invasive ILND along with the associated preliminary benefits.

## 2. Methods

We conducted an electronic systematic literature search of PubMed and Medline (OVID) identifying studies assessing surgical outcomes for open, laparoscopic and robotic ILND related to penile cancer. We included original studies published until April 2024. Key words included penile cancer/penile neoplasms, minimally-invasive procedures, robotics, video-endoscopic, robotic-assisted, inguinal lymphadenectomy, and inguinal lymph node dissections. We included controlled clinical studies, comparative studies, and randomized controlled trials. We excluded review articles, case reports, and studies that did not specifically evaluate penile cancer outcomes. We excluded articles that were not English. Our search focused on articles that specifically compared VEIL with open ILND outcomes yielding a total of 25 publication papers. Additional papers were added outside of the systematic literature review based on expert opinion. A narrative review of selected studies was performed.

## 3. Discussion

### 3.1 Patient selection and indications for surgery

ILND is an important component of the treatment pathway for select patients with penile cancer. Tumor pathological stage, lymphovascular invasion and >50% poorly differentiated cancer have been shown to be the strongest predictors of nodal metastasis [10]. According to the National Comprehensive Cancer Network (NCCN) guidelines, ILND is recommended in patients with high-risk disease (T1b or higher) with non-palpable inguinal nodes, high-risk disease with unilateral mobile nodes <4 cm and in patients with bulky or fixed nodes after neoadjuvant chemotherapy or if chemotherapy ineligible [11]. Inguinal lymph nodes are classified as superficial and deep. Superficial inguinal lymph nodes are located below the inguinal ligament and drain into scrotum, the anal canal (below the pectinate line), the skin below the umbilicus, and lower extremity. Deep inguinal lymph nodes are found within the femoral sheath medial to the femoral vein. Deep inguinal lymph nodes collect drainage from the glans penis, and the superficial lymph nodes. Both superficial and deep inguinal lymph nodes drain into the external iliac lymph nodes [12]. During ILND, superficial inguinal lymph nodes are removed first, and a specimen from the most concerning lymph node is sent to pathology for frozen section analysis. If a superficial node is positive for metastasis, deep inguinal lymph node dissection is performed. The fascia lata is opened and deep inguinal lymph nodes are dissected by following the saphenous vein to the femoral vein and skeletonizing the nodes to the node of Cloquet [13].

The use of dynamic sentinel node biopsy (DSNB) is another non-invasive approach to surgically stage nonpalpable inguinal lymph nodes (cN0) in patients with penile cancer. The use of ultrasound in this approach offers high clinical sensitivity rates up to 94% with low morbidity rates [14]. However, DSNB has shown to carry some concerning disadvantages including high-false negative rates and reduced accessibility in low-income countries or countries with high incidence of penile cancer. This could be attributed to high procedure costs or availability of nuclear department facilities especially when this technique is highly dependent on experienced nuclear medicine providers [15]. Even at designated cancer institutes, DSNB is not always available [2]. The current 2024 NCCN guidelines note the high false-negative rates concern with this MIS approach and therefore, recommend its use by only experienced physicians and tertiary centers that perform this procedure at least 20 times a year [11].

### 3.2 Surgical techniques

#### 3.2.1 Open ILND

The classic open ILND template involves removing nodal tissue bounded by the inguinal ligament superiorly, the adductor longus medially, the sartorius laterally and the fossa ovalis inferiorly. This template traditionally involved ligation of the greater saphenous vein and removal of the nodes deep to the fascia lata. The first modified template was described by Catalona *et al*. [16] in 1988 where all patients had resolved lymphedema postoperatively compared to over 50% of patients with debilitating lymphedema during the classic template era. Various other modified templates have been described to potentially lower post-operative complications [17, 18]. Spiess *et al*. [19] suggest an average rate of 19% and 27% in minor and major complications respectively in superficial ILND. While modified templates have been shown to have lower wound complication rates, they are recommended only for patients with increased risk for inguinal metastasis but a clinically negative groin exam. A full template dissection is recommended should nodal involvement be detected on frozen section during the initial modified template dissection [11].

#### 3.2.2 VEIL

**FIGURE 1.**
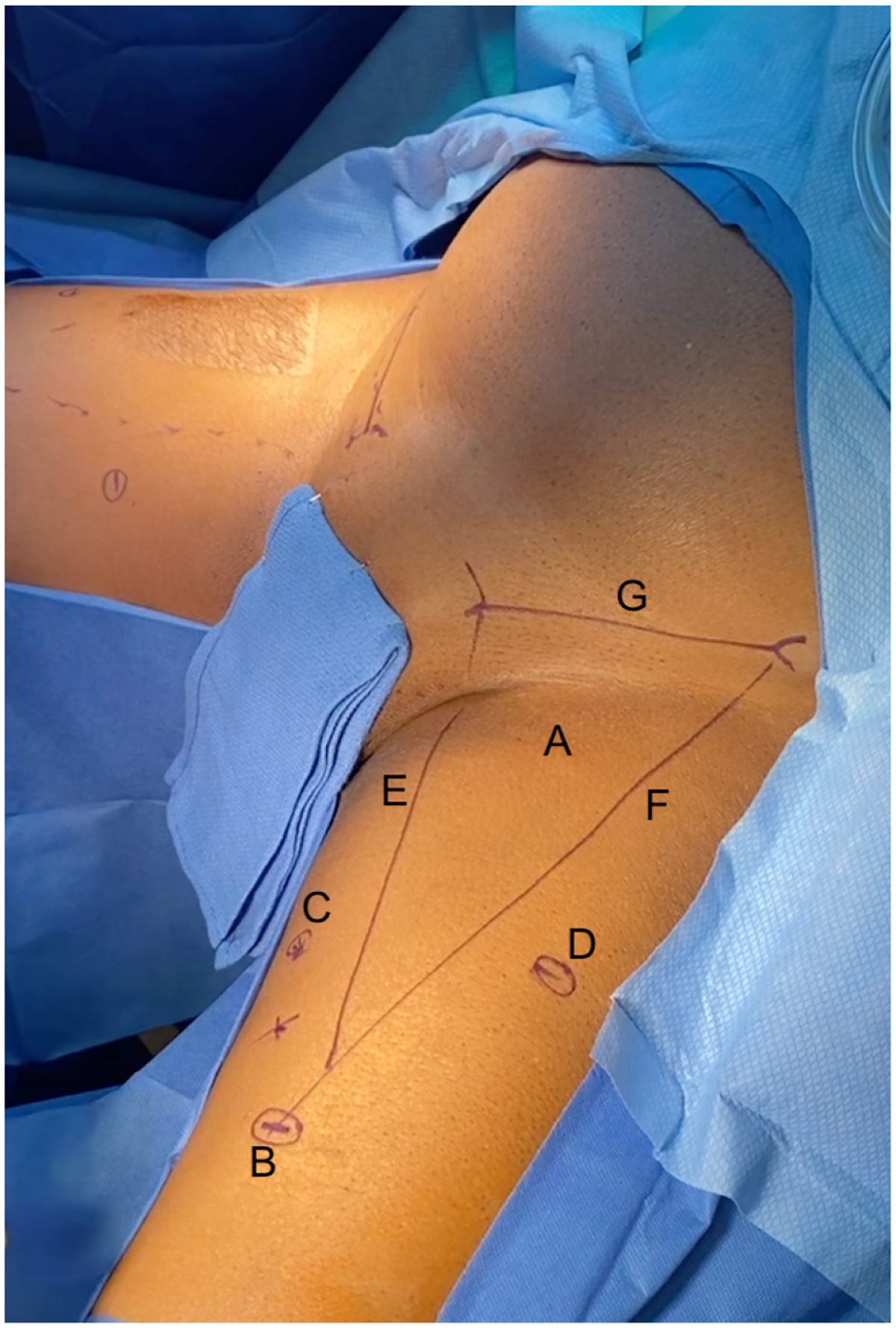
Incision outline in laparoscopic VEIL on left thigh. A 1.5 cm incision was made approximately 2 cm below the apex of the femoral triangle (A) to create a camera port (B). Medially and laterally to the camera port (~6 cm), 2 operative ports were placed (C,D, respectively). The borders of the femoral triangle are adductor longus muscle on the medial side (E), sartorius muscle on the lateral side (F), and inguinal ligament (G).

The VEIL approach mimics the same operative position and dissection boundaries as open ILND, incorporating the aforementioned modified template proposed by Catalona *et al*. [16]. This technique involves a 3-port access configuration distal to the femoral triangle; a camera port and 2 operative ports placed medially and laterally to the camera port (Fig. 1). After establishing a working space and identifying the saphenous vein, the superficial lymph nodes are dissected first before opening the femoral canal sheath to remove deep lymph nodes if indicated [20, 21].

#### 3.2.3 Robotic-VEIL surgical procedure

**FIGURE 2.**
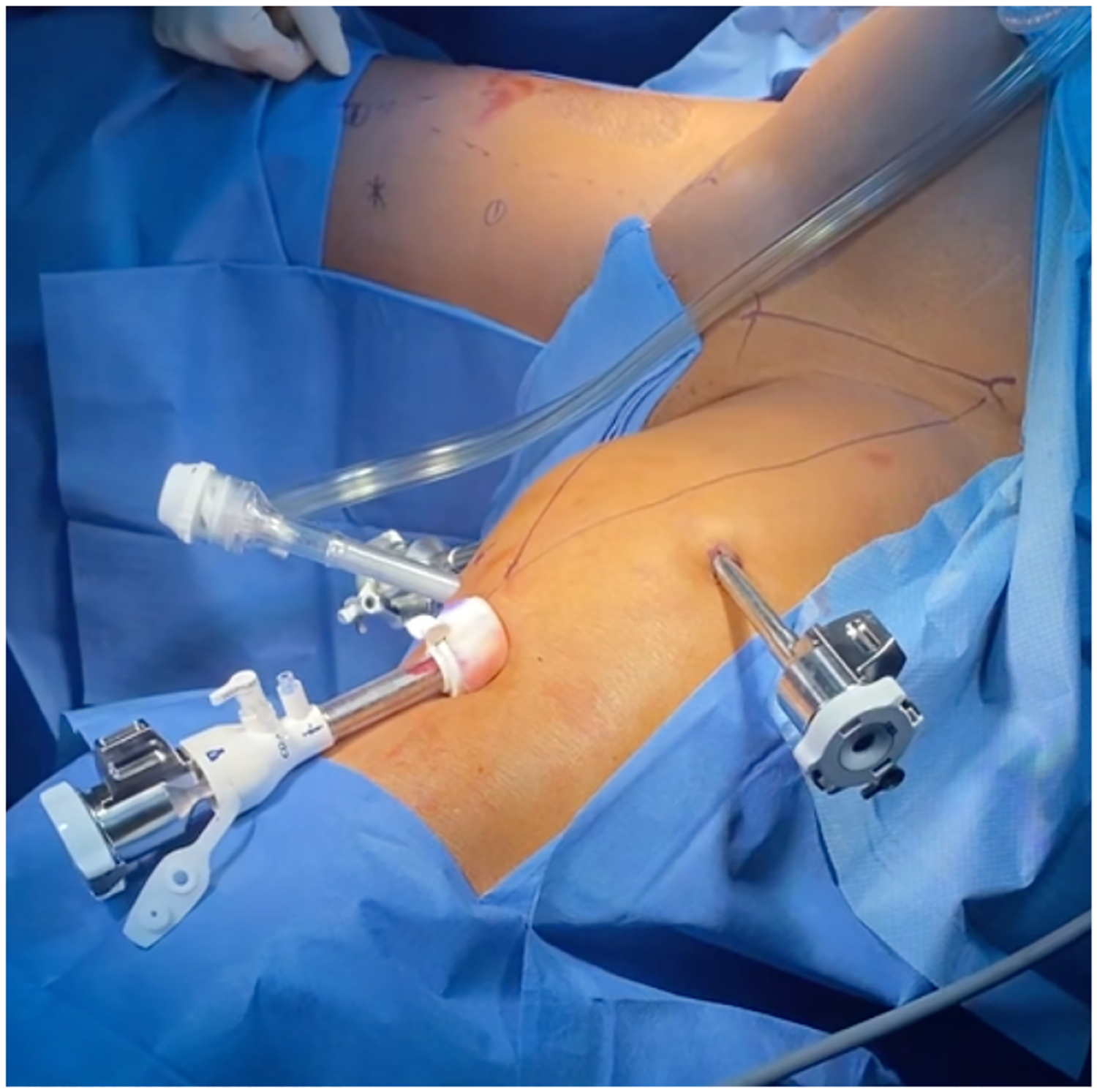
Robotic port placements in a patient undergoing robotic-VEIL. The robotic camera port was placed 25 cm inferior to the middle of the inguinal ligament. Two robotic ports were placed medially and laterally (~8 cm) from the camera port and an assistant port placed between the camera and medial robotic port.

Robotic-VEIL using Da Vinci Xi system has similar patient positioning, dissection outlines, and boundaries as laparoscopic VEIL with few modifications. The robotic camera port is placed inferior to the middle of the inguinal ligament, while the robotic arm ports are placed medially and laterally from the camera port outside the inguinal triangle. There is an assistant port that is placed between the camera and medial robotic arm as shown in Fig. 2. The superficial inguinal lymph node packet is separated from the fascia lata leaving it attached superiorly to scarpa fascia. The saphenous vein is preserved after careful clipping and transection of the accessory venous branches at the hilum. Lymphatics are often clipped to prevent post-operative lymphorrhea. Once the inguinal ligament is visualized along its entire length, the superficial lymph node packet is separated from the subcutaneous tissue and saphenous vein using cautery [13].

### 3.3 Surgical outcome advantages to MIS approach

VEIL and R-VEIL approaches to ILND are gaining popularity as newer techniques adapted from the original ILND. While limited randomized comparative studies exist, numerous cohort studies have demonstrated the safety and feasibility of VEIL [6, 21, 22]. Compared to open ILND, initial studies suggest an expected learning curve with VEIL reflected by longer operative times. The average operative times reported in open ILND studies ranged from 60 to 90 minutes [5, 21]. A retrospective, multicenter study by Tobias-Machado *et al*. [20] evaluated the surgical and oncological outcomes of VEIL in 105 patients with penile cancer. The average operative time was 90 minutes (60–120) with no conversion to open surgery, results similar to other studies that looked at operative time of VEIL [7, 21, 23].

A significant consequence of ILND regardless of approach is lymphedema. This can be mediated by careful clipping of lymphatics and preservation of the great saphenous vein (GSV) during the dissection [13]. Introducing a R-VEIL approach has been suggested to improve outcomes through more careful preservation of key structures due to better ergonomics, dexterity, and magnification compared to conventional open surgery [24]. GSV preservation can also be a factor impacting wound complications after ILND. In the original open approach to ILND, modified templates were introduced to enhance surgeons’ ability to preserve the GSV [7]. Ma *et al*. [25] conducted a retrospective study to evaluate the outcomes of partial preservation of GSV when dissecting inguinal lymph nodes in 182 patients with penile cancer. The authors concluded that a greater rate of preservation of the GSV and its branches during VEIL presented a significant advantage in reducing postoperative lower limb edema compared to open ILND. The feasibility of GSV sparing has been seen even more in R-VEIL compared to open ILND with better postoperative outcomes [25, 26]. In a retrospective study by Russell *et al*. [27], outcomes of VEIL and R-VEIL were analyzed in 18 penile cancer patients. Patients who underwent a R-VEIL had a 100% sparing of GSV rate compared with 57% in VEIL group, which is thought to be secondary to the increased flexibility of robotic arms [27, 28].

The length of hospital stay following ILND varies, however there was a significant difference based on approach; with most MIS techniques reporting around 2–3 days compared to 4–7 days in patients with open approach. In a prospective study by Kumar and retrospective study by Nayak, 42 and 39 patients with penile cancer respectively, were evaluated for postoperative complications including hospital stay between VEIL and open ILND approaches. The average hospital stay reported in Kumar and Nayak was 2.5 days and 3 days in VEIL approach, but 7.3 and 8 days in open ILND, respectively [29, 30].

Lymphorrhea is a common postoperative complication particularly seen in open ILND as a result of skin incisions in the groin compromising skin lymphatics. The smaller port incisions in the thigh instead of groin, preservation of skin lymphatics and vasculature, atraumatic retraction by gas, and avoidance of sartorius muscle rotation contribute to the reduced incidences of lymphorrhea and its associated complications seen in MIS approaches [5, 29]. In both open and MIS approaches, a percutaneous drain is often placed to mitigate lymphorrhea. The duration of indwelling drains varied between the different approaches. Sing *et al*. [5] reported a decreased drain time of 12 *vs*. 15 days with a lymphorrhea incidence of 0% *vs*. 9% in R-VEIL and open techniques, respectively. Nayak *et al*. [29] reported 100% *vs*. 8.7% of patients with removed drains by post-operative day 10 in VEIL and open ILND, respectively, and higher hospital readmissions due to associated lymphorrhea and drain complications in open compared to VEIL approach, 12 *vs*. 0, respectively.

Wound complications in MIS approaches were significantly less than open groups. A study of 92 open and 24 VEIL approaches yielded 47% flap necrosis complications and 24% wound dehiscence/infections compared to 4% and 0% respectively in the VEIL group [29]. Other studies suggest similarly significant decreases in wound complications. Yu *et al*. [28] retrospectively compared post-operative outcomes of 17 open and 20 RA-VEIL approach cases and reported 0% postoperative wound complications in the RA-VEIL group compared to 45% in the open group. Thyavihally *et al*. [7] found that wound complications were also linked to GSV preservation and pathological lymph node status. MIS techniques have been linked with low rate of high-grade complications (Clavien-Grade III or higher) in multiple studies; 1.9–10% in VEIL compared to a range of 17–68% in open approach cases [5, 20, 30].

Overall, MIS approaches to ILND have been suggested to decrease length of stay (LOS) for patients, decrease length of time for indwelling drain, lymphedema, and decrease wound complications namely infections or dehiscence [31]. A systemic review from 2019 corroborates that MIS approaches have been shown to decrease post-operative morbidity and decreased LOS (mean difference 1.77 days). ILND approach suggested increased rates of wound infection (odds ratio (OR) 10.62), lymphedema (OR 3.23), and Clavien-Dindo I–II complications (OR 4.58) and Clavien-Grade III–IV complications (OR 18.75) [31]. Randomized, prospective trials are needed to better define the true benefits on a larger scale.

A recently published randomized controlled trial (RCT) assessed ILND outcomes by comparing open ILND and VEIL approaches in 14 patients with penile cancer. Patients were randomized to receive open ILND or VEIL on one side, and then the opposite technique on the contralateral side. The VEIL approach showed significant reductions compared to open ILND approach in post-operative outcomes such as lymphedema rates 0% *vs*. 35.7% respectively, drain removal (15 days *vs*. 27 days, respectively), and leg discomfort (0% *vs*. 28.6%, respectively). There were no significant differences in node count or operative time between the two groups [32].

### 3.4 Oncologic benefits

Studies comparing MIS and open approaches shown no significant difference in oncologic outcomes in terms of recurrence and survival rates. However, MIS approaches have been associated with higher node count which while not a direct surrogate for oncologic outcomes is noteworthy [33]. Ji *et al*. [22] compared the outcomes between R-VEIL and VEIL and concluded the mean number of lymph nodes resected was 22.2 and 15.4 in R-VEIL and VEIL groups, respectively. The robotic approach is thought to offer a more precise motion for dissection of smaller compared to the standard open approach which is particularly helpful to safely and completely remove inguinal lymph nodes [22]. However, the true oncologic benefits of MIS approaches to ILND have yet to be established.

### 3.5 Limitations of the minimally invasive approach

One of the most universally accepted disadvantages of R-VEIL compared to either open ILND or VEIL is the financial burden on both patients and the healthcare system. Since the R-VEIL era is still on a learning curve, it is challenging to accept an expensive method with a high learning curve to show superiority in terms of oncological and surgical benefits over traditional approaches [2]. Similarly, given the limited volume of penile cancer in combination with the necessary learning curve to master MIS approaches, it may take some time before a provider experiences the improved outcomes discussed in this review [20]. However, while the cost to perform a RA-VEIL procedure is drastically higher than an open ILND procedure, this can be offset by saving costs associated with less hospital stay and management of complications [5].

Several limitations and biases are worth noting in our study. The relatively small sample sizes identified in the extracted articles, lack of long-term follow up outcomes [33], and limiting the scope of MIS in only penile cancer, are all essential factors that make it challenging to generalize and confidently adopt its technique [31]. Additionally, data on oncological outcomes such as survival and recurrence rates in patients with specific nodal status was limited, thus it is too early to definitively deem one technique superior oncologically.

While there are multiple studies that suggest lower rate of post-operative complications utilizing MIS techniques to ILND, these are predominately retrospective in nature with concern for selection bias. Furthermore, the heterogeneity found within included studies and inconsistency of reported outcome variables and analysis introduces publication and reporting bias [23]. Prior to determining the true benefits of MIS ILND from both a complications and oncologic perspective, more randomized control trials need to be performed. However, the paucity of patients undergoing ILND for penile cancer creates a barrier to quality prospective studies.

## 4. Conclusion

In conclusion, the management of penile cancer involving ILND is undergoing a transformative shift with the emergence of MIS techniques, specifically VEIL and R-VEIL. While traditional open ILND has been the gold standard, it is often associated with substantial morbidity, particularly high wound complications. The advantage of MIS approach aims to mitigate these challenges and enhance overall patient outcomes. Reductions in postoperative complications such as lymphedema are seen more when sparing the great saphenous vein. Comparative analyses suggest that MIS approaches exhibit favorable outcomes, including shorter hospital stays, reduced drain times, and decreased postoperative complications, and improved wound healing [5, 20, 28–31]. Current literature, although promising, is predominantly retrospective, necessitating randomized controlled trials for a more comprehensive understanding of both the post-operative and oncologic benefits of MIS approaches to ILND.

## ABBREVIATIONS

ILND: inguinal lymph node dissection
MIS: minimally invasive surgical
VEIL: video-endoscopic inguinal lymphadenectomy
R-VEIL: robotic-assisted video-endoscopic inguinal lymphadenectomy
